# Supplementary material for: Systematic review of residual toxicity studies of pesticides to bees and veracity of guidance on pesticide labels
Source: PeerJ. 2024 Jan 3;12:e16672. doi: 10.7717/peerj.16672 (PMC10771081; doi:10.7717/peerj.16672)
Supplement: Supplemental Information 2 [file peerj-12-16672-s002.docx]

| **Section and Topic** | **Item #** | **Checklist item** | **Reported on page** |
| --- | --- | --- | --- |
| **TITLE** | | |  |
| Title | 1 | Identify the report as a systematic review. | 1 |
| **ABSTRACT** | | |  |
| Abstract | 2 | See the PRISMA 2020 for Abstracts checklist. | 2-3 |
| **INTRODUCTION** | | |  |
| Rationale | 3 | Residual toxicity values are used by pesticide applicators and regulators to protect bees. There is no comprehensive database of residual toxicity values to check residual toxicity statements on pesticide labels. Also, there has not be a recent review of residual toxicity values in the literature. | 2-4 |
| Objectives | 4 | “Our objective was to provide the first analysis of pesticide label statements communicating residual toxicity to bees in comparison to actual RT_25_ values” | 4 |
| **METHODS** | | |  |
| Eligibility criteria | 5 | “We located putative residual toxicity studies with the search term “residual toxicity” as well as the names of bee taxa commonly used in residual toxicity assays and currently listed in EPA’s RT_25_ database: “*Apis*”, “*Nomia*” and “*Megachile*”.” | 5 |
| Information sources | 6 | Web of Science. Proceedings of the Western Alfalfa Seed Growers. Bee Research Investigation and Integrated Pest and Pollinator Investigation reports released by Washington State University. Bayer CropSciences. | 6 |
| Search strategy | 7 | Present the full search strategies for all databases, registers and websites, including any filters and limits used. | 6 |
| Selection process | 8 | “We narrowed these studies down to 50 by only including studies that met any of the following criteria: (1) the study was a primary source of data (*e.g*., not a review paper); (2) bees were exposed to the pesticide applied to on plant material (e.g., no studies where pesticide was applied to filter paper); and/or, (3) the study focused on residual toxicity of pesticides applied to plots of crop plants and involved harvesting plant tissue for caged bees. These criteria were designed to ensure that we only included studies whose residual toxicity methodology broadly followed those of EPA (2016).” | 6 |
| Data collection process | 9 | Lead author collected and reviewed studies. | 6 |
| Data items | 10a | Pesticide rate and formulation and endpoints were the percentage of bees dead at different periods after exposure. | 7 |
|  | 10b | Test conditions | 6 |
| Study risk of bias assessment | 11 | No bias expected | N/A |
| Effect measures | 12 | RT25 values (defined in manuscript) | 7 |
| Synthesis methods | 13a | Describe the processes used to decide which studies were eligible for each synthesis (e.g. tabulating the study intervention characteristics and comparing against the planned groups for each synthesis (item #5)). | N/A |
|  | 13b | We removed two additional studies because the author indicated that it was likely that some live bees in the assay were mistakenly counted as dead (Johansen *et al*., 1981) and because the actual active ingredient of the product used was not specified (Walsh, 2011). | 6 |
|  | 13c | Describe any methods used to tabulate or visually display results of individual studies and syntheses. | Supplementary Data |
|  | 13d | Describe any methods used to synthesize results and provide a rationale for the choice(s). If meta-analysis was performed, describe the model(s), method(s) to identify the presence and extent of statistical heterogeneity, and software package(s) used. | N/A |
|  | 13e | Describe any methods used to explore possible causes of heterogeneity among study results (e.g. subgroup analysis, meta-regression). | N/A |
|  | 13f | Describe any sensitivity analyses conducted to assess robustness of the synthesized results. | N/A |
| Reporting bias assessment | 14 | We used the following approaches to standardize methodologies across studies. EPA uses the word “young” to describe the optimal age of bees for residual toxicity trials. We interpreted “young” to mean newly emerged adult (eclosed) bees that were less than 1 day of age (Winston, 1991). Furthermore, when a study reported a range for a parameter, such as for number of bees per cage or temperature, we used the average calculated from the low and high points of the range. In reference to the diet that the bees were fed during the assay, one study reported the syrup concentration as 91:1 (wt:wt) which we assumed was 1:1 (Mayer, 2001). | 7 |
| Certainty assessment | 15 | We assumed EPA database estimates were accurate and compared correspondence. | 8 |
| **RESULTS** | | |  |
| Study selection | 16a | Describe the results of the search and selection process, from the number of records identified in the search to the number of studies included in the review, ideally using a flow diagram. | See Prisma Flow Diagram |
|  | 16b | We removed two additional studies because the author indicated that it was likely that some live bees in the assay were mistakenly counted as dead (Johansen *et al*., 1981) and because the actual active ingredient of the product used was not specified (Walsh, 2011). | 6 |
| Study characteristics | 17 | Cite each included study and present its characteristics. | Supplementary Data Material |
| Risk of bias in studies | 18 | Present assessments of risk of bias for each included study. | N/A |
| Results of individual studies | 19 | For all outcomes, present, for each study: (a) summary statistics for each group (where appropriate) and (b) an effect estimate and its precision (e.g. confidence/credible interval), ideally using structured tables or plots. | Table 2 |
| Results of syntheses | 20a | For each synthesis, briefly summarise the characteristics and risk of bias among contributing studies. | N/A |
|  | 20b | Present results of all statistical syntheses conducted. If meta-analysis was done, present for each the summary estimate and its precision (e.g. confidence/credible interval) and measures of statistical heterogeneity. If comparing groups, describe the direction of the effect. | Table 2 Fig 1-5 |
|  | 20c | Present results of all investigations of possible causes of heterogeneity among study results. | 11-12 |
|  | 20d | Present results of all sensitivity analyses conducted to assess the robustness of the synthesized results. | 11-12 |
| Reporting biases | 21 | Present assessments of risk of bias due to missing results (arising from reporting biases) for each synthesis assessed. | N/A |
| Certainty of evidence | 22 | Present assessments of certainty (or confidence) in the body of evidence for each outcome assessed. | Table 2  Fig 1-5 |
| **DISCUSSION** | | |  |
| Discussion | 23a | Provide a general interpretation of the results in the context of other evidence. | 12-18 |
|  | 23b | Discuss any limitations of the evidence included in the review. | 12-18 |
|  | 23c | Discuss any limitations of the review processes used. | 17 |
|  | 23d | Discuss implications of the results for practice, policy, and future research. | 18 |
| **OTHER INFORMATION** | | |  |
| Registration and protocol | 24a | Provide registration information for the review, including register name and registration number, or state that the review was not registered. | Not registered |
|  | 24b | Indicate where the review protocol can be accessed, or state that a protocol was not prepared. | State protocol not prepared |
|  | 24c | Describe and explain any amendments to information provided at registration or in the protocol. | N/A |
| Support | 25 | Describe sources of financial or non-financial support for the review, and the role of the funders or sponsors in the review. | 19 |
| Competing interests | 26 | Declare any competing interests of review authors. | PeerJ form |
| Availability of data, code and other materials | 27 | Report which of the following are publicly available and where they can be found: template data collection forms; data extracted from included studies; data used for all analyses; analytic code; any other materials used in the review. | Supplementary Materials. |

*From:*  Page MJ, McKenzie JE, Bossuyt PM, Boutron I, Hoffmann TC, Mulrow CD, et al. The PRISMA 2020 statement: an updated guideline for reporting systematic reviews. BMJ 2021;372:n71. doi: 10.1136/bmj.n71

For more information, visit: <http://www.prisma-statement.org/>
